# Endoscopic and clinicopathological features of intramucosal, histologically mixed-type, low-grade, well-differentiated gastric tubular adenocarcinoma with the potential for late-onset lymph node metastasis

**DOI:** 10.1186/s12876-018-0919-3

**Published:** 2018-12-27

**Authors:** Takashi Saitoh, Asako Takamura, Gen Watanabe

**Affiliations:** 1 Division of Gastrointestinal Endoscopy and Gastroenterology, Niigata Prefectural Kamo Hospital, 1-9-1 Aomi-cho, Kamo, Niigata, 959-1397 Japan; 2 Department of Gastrointestinal Endoscopy and Gastroenterology, Niigata Prefectural Federation of Japan Agricultural Cooperatives for Health and Welfare, Toyosaka Hospital, 1-11-1 Isurugi, Kita-ku, Niigata, 950-3327 Japan; 3 Department of Pathology, Niigata Cancer Center Hospital, 2-15-3 Kawagishi-cho, Chuo-ku, Niigata, 951-8566 Japan; 4 Division of Molecular and Diagnostic Pathology, Niigata University Graduate School of Medical and Dental Sciences, 1-757 Asahimachi-dori, Chuo-ku, Niigata, 951-8510 Japan

**Keywords:** Early gastric cancer, Premalignant stomach neoplasms, Low-grade, well-differentiated tubular adenocarcinoma, Undifferentiated-type cancer components, Gastric mucins/phenotype, Endoscopic submucosal dissection, Narrow band imaging, Magnifying endoscopy, *Helicobacter pylori* - eradication

## Abstract

**Background:**

Intramucosal, histologically mixed-type, low-grade (LG), well-differentiated gastric tubular adenocarcinomas (tub1s; LG-tub1s) have larger mean diameters and exhibit a higher frequency of the gastric mucin phenotype (G-phenotype) than pure LG-tub1s. In proportion to their increases in diameter, G-phenotype differentiated-type early gastric cancer (EGC) tumours reportedly grow to eventually contain (an) undifferentiated-type component(s) and LG-tub1s, which are included in differentiated-type EGCs, reportedly exhibit changes in their glandular architectural and cytological atypia grades from LG to high-grade (HG) and can grow to contain a moderately differentiated tubular adenocarcinoma (tub2) component and undifferentiated components. Because they generally show a higher frequency of malignancy relative to tumours with a higher atypia grade and lower differentiation degree, it is suggested that, among mixed-type LG-tub1s, G-phenotype LG-tub1s containing an HG-tub2 component (LG-tub1s > HG-tub2) with undifferentiated components might lead to late-onset metastasis to lymph nodes even after a successful endoscopic submucosal dissection (ESD). We aimed to clarify the endoscopic and clinicopathological features of these G-phenotype LG-tub1s > HG-tub2.

**Methods:**

Of the 13,217 oesophagogastroduodenoscopies performed at our institutions between September 2008 and March 2016, 185 EGC lesions were evaluated in this retrospective observational study. Among these EGC lesions, 60 intramucosal LG-tub1s were divided into 53 tub1 (44 pure LG-tub1s and nine LG-tub1s containing HG-tub1) lesions and seven LG-tub1 > tub2 (LG-tub1 containing LG- and HG-tub2) lesions.

**Results:**

The frequencies of the superficial depressed type (*P* = 0.026), reddish colour (*P* = 0.006), HG of contained tub2s (*P* = 0.006), and G-phenotype (*P* = 0.028) were significantly higher in the LG-tub1 > tub2 group than those in the tub1 group. However, the largest lesion of the LG-tub1 > tub2 group had a superficial flat appearance, an isochromatic colour, an HG-tub2 and an undifferentiated component, and a large diameter greater than 30 mm, and it exhibited a G-phenotype.

**Conclusions:**

Intramucosal G-phenotype LG-tub1s > HG-tub2 are potential premalignant stomach neoplasms that may have specific endoscopic and clinicopathological features. However, G-phenotype LG-tub1s > HG-tub2 with undifferentiated component, which potentially show higher malignancy than those without undifferentiated components might change from a reddish to isochromatic colour. Accurately diagnosing, treating, and following-up G-phenotype LG-tub1s > HG-tub2 might decrease the number of patients who experience late-onset metastasis after ESD.

**Electronic supplementary material:**

The online version of this article (10.1186/s12876-018-0919-3) contains supplementary material, which is available to authorized users.

## Background

Gastric cancer is classified as differentiated- or undifferentiated-type cancer (synonymous with intestinal- and diffuse-type cancer, respectively) [1–3]. Early gastric cancer (EGC) is confined to the mucosal or submucosal layer, irrespective of lymph node metastasis [3]. Intramucosal, low-grade (LG), well-differentiated tubular adenocarcinomas (tub1; LG-tub1s) have been categorized as a differentiated-type EGCs and are assigned to category 4 according to the revised Vienna classification system (Table 1) [4–6]. Tumours that are diagnosed as LG-tub1 by Japanese gastrointestinal pathologists may be diagnosed as high-grade dysplasia by Western gastrointestinal pathologists (Table 2) and are reportedly associated with submucosal invasion and lymphatic permeation [7].

**Table 1:** Histological subtypes of differentiated-type and undifferentiated-type of EGC [3]

Type of differentiation	Histological subtype	Remarks
Differentiated	Well-differentiated tubular adenocarcinoma (tub1)	Intramucosal tumours of this type have been assigned to category 4 according to the revised Vienna classification system [4–6].
	Moderately differentiated tubular adenocarcinoma (tub2)	
	Papillary adenocarcinoma (pap)	This type of tumour and component was not included in any of the enrolled EGCs in this study unintentionally.
Undifferentiated	Solid type, poorly differentiated adenocarcinoma (por1)	
	Non-solid type, poorly differentiated adenocarcinoma (por2)	
	Signet-ring cell carcinoma (sig)	
	Mucinous adenocarcinoma (muc)	

*EGC* early gastric cancer

**Table 2:** Relationship between atypia grades and histological subtypes of differentiated-type EGC [3, 4, 8]

Atypia grade	Histological subtype		Remarks
	Tub1	Tub2	
Low-grade (LG)	LG-tub1	LG-tub2	Tumours that are diagnosed as LG-tub1 by Japanese gastrointestinal pathologists may be
High-grade (HG)	HG-tub1	HG-tub2	diagnosed as high-grade dysplasia by Western gastrointestinal pathologists [7].

*EGC* early gastric cancer; *LG-tub1* low-grade, well-differentiated tubular adenocarcinoma; *LG-tub2* low-grade, moderately differentiated tubular adeno-carcinoma; *HG-tub1* high-grade, well-differentiated tubular adenocarcinoma; *HG-tub2* high-grade, moderately differentiated tubular adenocarcinoma

Because these tumours occasionally have an isochromatic colour under white light endoscopy (WLE) and unclear demarcation lines (DLs) not only under WLE but also under narrow-band imaging with magnifying endoscopy (NBI-ME), they are reportedly difficult to diagnose, which may result in an eventual increase in their diameters [8]. We have also reported that intramucosal histologically mixed-type LG-tub1 lesions, which predominantly consist of LG-tub1s, minor high-grade (HG) tub1s and/or minor moderately differentiated tubular adenocarcinomas (tub2s) and/or (a) minor undifferentiated component(s), have larger mean diameters and exhibit a higher frequency of a gastric mucin phenotype (G-phenotype) than pure LG-tub1 lesions; thus, they may be primitive lesions that precede malignant transformation [8]. However, the atypia grades and histological subtypes that exist in these intramucosal G-phenotype histologically mixed-type LG-tub1 lesions have not yet reported. In proportion to tumour diameter, possible changes have been reported in the degree of differentiation of G-phenotype differentiated-type EGC, e.g., changes from tub2 to tub2 containing (an) undifferentiated component(s) [9–13]. Also in proportion to diameter, possible changes in the glandular architectural and cytological atypia grades from LG to HG have been reported [4, 8] in differentiated-type EGC lesions, including in mixed-type LG-tub1s, e.g., changes from LG-tub1 to HG-tub1. Therefore, it is suggested that LG-tub1s containing HG-tub1 (LG-tub1s > HG-tub1) can potentially become LG-tub1s containing HG-tub2 (LG-tub1s > HG-tub2) without (a) minor undifferentiated component(s). A carcinoma with a higher atypia grade and lower differentiation degree exhibit higher malignancy, and LG-tub1 > HG-tub2 lesions with (a) minor undifferentiated component(s) are the most malignant lesions among the histologically pure and mixed-type LG-tub1 lesions. Intramucosal G-phenotype LG-tub1s > HG-tub2 without (a) minor undifferentiated component(s) have been suggested to grow to contain (a) minor undifferentiated component(s) and result in lymph node metastasis, whereas G-phenotype LG-tub1 lesions containing other minor differentiated-type components of other atypia grades and subtypes may not do so [8–13].

Differentiated-type predominant, histologically mixed-type EGC is generally defined as EGC that consists of predominant differentiated-type components containing (a) minor undifferentiated component(s) that has (have) smaller diameters than those of the predominant components. In Japanese gastric cancer treatment guidelines, endoscopic submucosal dissection (ESD) is a treatment applied for EGC of absolute and expanded indications [14]. Nearly all patients who undergo curative resection for intramucosal EGC lesions have a good prognosis [15–17]. However, the evidence is insufficient regarding whether intramucosal, G-phenotype LG-tub1 > HG-tub2 lesions that have diameters of more than 30 mm, and that have diameters of more than 40 mm and contain (a) minor undifferentiated component(s) whose diameters are more than 20 mm, can be cured by ESD. [14, 18–21]. In some of these intramucosal G-phenotype LG-tub1 > HG-tub2 lesions that have diameters of more than 40 mm and that contain (a) minor undifferentiated component(s), determining the diameters of the minor undifferentiated component(s) is occasionally difficult due to the difficult histological diagnosis [3]. These types of intramucosal G-phenotype LG-tub1 > HG-tub2 lesions that have diameters of more than 30 mm and those that have diameters of more than 40 mm containing (a) minor undifferentiated component(s) whose diameters are more than 20 mm, are occasionally observed and may be resected via ESD after diagnosis as intramucosal differentiated-type EGCs that meets the expanded indication for ESD. However, after ESD and detailed histopathological examination, these tumours might be found to not meet the current curative criteria for ESD and have a potential for late-onset lymph node metastasis, resulting in death. [14, 18–21].

Therefore, accurately diagnosing and treating G-phenotype LG-tub1 > HG-tub2 lesions, especially those with diameters of more than 30 mm and those with diameters of more than 40 mm containing (a) minor undifferentiated component(s) whose diameters are more than 20 mm, may be useful for patients with these lesions, help clinicians carefully follow up after ESD and might decrease the number of patients who experience late-onset lymph node metastasis.

Consequently, to clarify the clinicopathological features of these G-phenotype LG-tub1s > HG-tub2 is potentially important for gastric cancer screening.

## Methods

### Patients and inclusion/exclusion criteria

Lesions were included if they met the following criteria: (1) biopsy-diagnosed differentiated-type EGC lesions that included an LG-tub1 component and (2) consecutive differentiated-type EGC lesions within the absolute or expanded ESD indications that were treated with current curative resections via ESD at one of the participating hospitals and were histopathologically found to include a predominant LG-tub1 component [8, 14]. Lesions were excluded if the EGC that was diagnosed post-ESD through histological examination met any of the following criteria: (1) the lesion did not have a predominant LG-tub1 component, (2) there was evidence of submucosal invasion, or (3) degenerated areas resulting from the burning effect of ESD were present, which would make detailed comparisons with the endoscopic findings difficult.

**Fig. 1:**
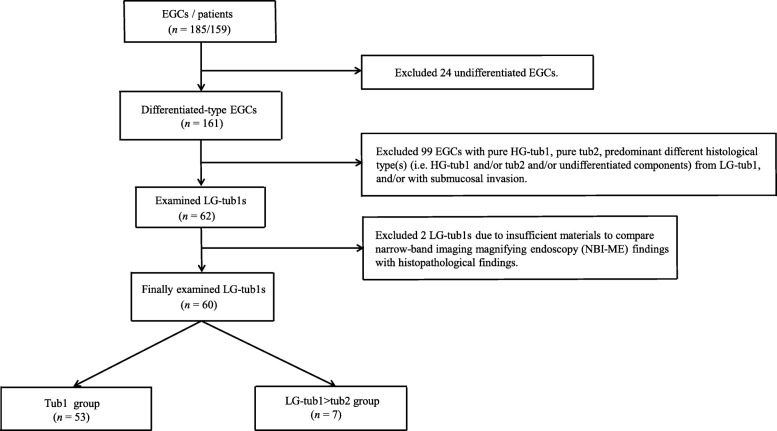
Study flowchart. EGC, early gastric cancer; HG-tub1, high-grade, well-differentiated tubular adenocarcinoma; tub2, moderately differentiated tubular adenocarcinoma; LG-tub1(s), low-grade, well-differentiated tubular adenocarcinoma(s); LG-tub1 > tub2, LG-tub1 containing LG-tub2 or HG-tub2

Of the 13,217 patients who underwent oesophagogastroduodenoscopy at our institutions between September 2008 and March 2016, 185 EGC lesions (161 differentiated-type and 24 undifferentiated-type lesions) from 159 patients were evaluated in this retrospective study (Fig. 1). Of the 161 differentiated-type EGC lesions, 99 were excluded because they did not include an LG-tub1 component or predominantly consisted of a different histological component(s). An additional two lesions were excluded because the post-ESD specimens exhibited partial degeneration due to the burning effect of ESD.

The present study was conducted in accordance with the Declaration of Helsinki and approved by the Ethics Committee of Kamo Hospital and by the Ethical Review Board for Clinical Research of Toyosaka Hospital. Reference numbers were not assigned. Prior to the first endoscopy, written informed consent was obtained from each patient included in the study.

### Endoscopy

All the endoscopic diagnostic procedures were performed by two highly experienced endoscopists (certified by the Japan Gastroenterological Endoscopy Society) using a high-resolution upper gastrointestinal endoscope (GIF-H260, Olympus Medical Systems, Tokyo, Japan), a magnifying upper gastrointestinal endoscope (GIF-Q260Z, Olympus Medical Systems), and two electronic endoscopy systems (EVIS LUCERA Spectrum and EVIS LUCERA ELITE Spectrum; Olympus Medical Systems). NBI-ME was performed using a GIF-Q260Z endoscope fitted with a soft black hood (MB-46, Olympus Medical Systems) by the same endoscopist who performed ESD [22].

### Cancer diagnostic procedure

The lesions of interest were diagnosed as cancerous or non-cancerous, as advanced cancer or EGC, and as differentiated- or undifferentiated-type EGC based on WLE with chromoendoscopy and NBI-ME. High-resolution WLE and NBI-ME images collected before and after ESD were stored on the hard discs of the endoscopy systems. The diameter, macroscopic type, location, predominant colour on WLE, DL on NBI-ME, LG or HG type, and background mucosal atrophy according to the Kimura-Takemoto classification of each lesion of interest were examined [3, 8, 23, 24]. The degree of background mucosal atrophy in this study was defined as mild (C-I and C-II), moderate (C-III and O-I), or severe (O-II and O-III). The absence of an ulceration in the lesion, the indication for ESD, and whether the lesion was metachronous (detected > 1 year after successful ESD and/or endoscopic mucosal resection) were determined. The NBI-ME findings for all the lesions were retrospectively reviewed for a limited-to-four-pattern sign and were collated using a magnifying endoscopy simple diagnostic algorithm for EGC (MESDA-G) by highly experienced endoscopists who were blinded to the prior histopathological diagnoses [8, 23].

### *Helicobacter pylori* status

Anti-*H. pylori* immunoglobulin G was assayed in the urine (Otsuka Pharmaceutical Co. Ltd., Tokyo, Japan) and in the serum (Eiken Chemical Co. Ltd., Tokyo, Japan), and rapid urease and urea breath tests were performed to determine the presence of *H. pylori*. Patients with positive results on any test were considered positive for *H. pylori*, whereas those with negative results on at least two of the three tests were considered negative for *H. pylori*. Whether *H. pylori* had been eradicated for > 1 year prior to the present study was determined.

### Endoscopic submucosal dissection (ESD)

ESD was performed by a single endoscopist skilled in ESD using a GIF-Q260Z endoscope. To enable detailed comparison with the post-removal histopathological findings, cautery marks were made around the lesions, and images were captured before and after ESD with WLE and NBI-ME.

### Histopathological examination

The post-ESD specimens were prepared for histopathological examination. The maximum and minimum diameters were measured. The specimens were fixed in formalin (10%) and embedded in paraffin, and thin sections were stained with haematoxylin-eosin (HE) and antibodies against CD10, MUC2, MUC6, MUC5AC, and (p53) (Novocastra, Newcastle, UK); and CDX2 and Ki-67 (Dako Japan, Tokyo, Japan). All histopathological diagnoses were made by a highly experienced gastrointestinal pathologist certified by the Japanese Society of Pathology. Atypia grades were assigned as previously described [4, 8]. Staining for the proliferative capacity of the carcinoma cells (Ki-67) and a tumour suppressor gene (p53) served as references for the determination of the atypia grade [8]. All the intramucosal LG-tub1 lesions were classified as tub1 if they consisted of pure LG-tub1 or LG-tub1 > HG-tub1 or were classified as LG-tub1 > tub2 if they consisted of LG-tub1 containing LG-tub2 or HG-tub2. All the post-ESD lesions were immunohistochemically characterized as one of four mucin phenotypes: gastric (G), gastrointestinal (GI), intestinal (I), or null (N) [25].

### Assessed parameters

The primary study endpoint was the difference in the frequencies/ratios of the clinicopathological parameters between the tub1 and LG-tub1 > tub2 groups. The secondary study endpoints were the between-group differences in the clinically significant clinicopathological parameters.

### Statistical analysis

All statistical analyses were performed using SPSS software, version 24.0 (IBM Japan, Tokyo, Japan). The continuous variables (patient age and maximum lesion diameter) in the tub1 and LG-tub1 > tub2 groups were compared using Student’s *t*-tests. The intergroup differences in the categorical variables (sex, macroscopic type, location, predominant colour, determination of a DL on NBI-ME, background mucosal atrophy, metachronous occurrence > 1 year after successful ESD and/or endoscopic mucosal resection, *H. pylori* status, diagnosis of LG or HG, and mucin phenotype) were determined using a chi-squared test, Fisher’s exact test, or likelihood ratio test as appropriate. Significantly different factors were subjected to a multivariate logistic regression analysis. *P* values < 0.05 were considered statistically significant.

## Results

### Endoscopic and histopathological findings

Histopathological examination of the 60 included LG-tub1 lesions after ESD revealed that 53 lesions were classified into the tub1 group, with 44 pure LG-tub1 lesions and nine LG-tub1 > HG-tub1 lesions. Among the 7 lesions in the LG-tub1 > tub2 group, 1 was an LG-tub1 lesion containing LG-tub2 (LG-tub1 > LG-tub2) and 6 were LG-tub1 > HG-tub2 lesions (Table 3). In addition, seven of the 60 examined lesions showed partly unclear DLs even on NBI-ME, belonged to the tub1 group, and consisted of four pure LG-tub1 and three LG-tub1 > HG-tub1 lesions. The four pure LG-tub1 lesions were classified into one G-phenotype, one GI-phenotype, and two I-phenotype. The three LG-tub1 > HG-tub1 lesions were classified into one G-phenotype and two GI-phenotype. The two GI-phenotype LG-tub1 > HG-tub1 lesions were larger than 30 mm.

**Table 3:** Characteristics of the patients in the tub1 and LG-tub1 > tub2 groups

Group (Number of lesions)	Tub1 group (*n* = 53)	LG-tub1 > tub2 group (*n* = 7)	*P* value
Age, mean ± SD years (range)	73.3 ± 7.3 (50–91)	73.7 ± 11.9 (57–93)	0.946
Sex (male/female)	36/17	6/1	0.663
Diameter, mean ± SD mm (range)	11.5 ± 10.7 (2–70)	15.3 ± 7.6 (8–31)	0.368
Macroscopic type			
0-IIa/0-IIb/0-IIc^a^	36/9/8	1/1/5	0.026^★^
Colour on WLE			
Whitish/isochromatic/reddish	36/9/8	1/1/5	0.006^★^
DL on NBI-ME			
Clear/partly unclear	46/7	7/0	0.584
Cytological and glandular architectural atypia grade			
Low-grade/high-grade	44/9	1/6	0.001^★^
Background mucosal atrophy			
C-I, II/C-III, O-I/-II, III^b^	0/5/48	0/0/7	1.000
Endoscopic resected metachronous EGC lesion			
Yes/No	2/51	3/4	0.009^★^
*H. pylori* status			
Positive/negative/eradicated	24/23/6	5/2/0	0.269
Mucin phenotype			
Gastric /gastrointestinal/intestinal	6/19/28	4/1/2	0.028^★^

*Tub1* well-differentiated tubular adenocarcinoma; *LG* low-grade; *Tub2* moderately differentiated tubular adenocarcinoma; *LG-tub1 > tub2* LG-tub1 containing tub2; *SD* standard deviation; *WLE* white light endoscopy; *DL* demarcation line; *NBI-ME* narrow-band imaging with magnifying endoscopy; *EGC* early gastric cancer; ^a^*0-IIa* superficial elevated; *0-IIb* superficial flat; *0-IIc* superficial depressed type [3]; ^b^*Kimura-Takemoto classification* [24] ^★^*P* < 0.05. Continuous variables were compared using Student’s *t*-tests. Intergroup differences in the categorical variables were determined using the chi-squared test, Fisher’s exact test, or a likelihood ratio test as appropriate

There were no significant differences between the tub1 group and the LG-tub1 > tub2 group in terms of the maximum tumour diameter, location, DL on NBI-ME, or degree of background mucosal atrophy. The frequencies of the superficial depressed (0-IIc) type (*P* = 0.026), a reddish colour (*P* = 0.006), HG histological components (*P* = 0.001), and the G-phenotype (*P* = 0.028) were significantly higher in the LG-tub1 > tub2 group than those in the tub1 group. The groups, types of LG-tub1s, contained components, abbreviations, illustrations, colour, and mucin phenotype of a superficial elevated type (0-IIa), I-phenotype pure LG-tub1 lesion (Additional file 1: Figure S1); a superficial flat type (0-IIb), GI-phenotype, LG-tub1 > HG-tub1 lesion (Additional file 2: Figure S2); a superficial shallow depressed type (0-IIc), I-phenotype, LG-tub1 > LG-tub2 lesion (Additional file 3: Figure S3); a superficial depressed type (0-IIc), G-phenotype LG-tub1 > HG-tub2 lesion (Additional file 4: Figure S4); and a superficial flat type (0-IIb), G-phenotype LG-tub1 > HG-tub2 lesion (Additional file 5: Figure S5) are presented in Table 4.

**Table 4:** Histologically pure LG-tub1 and mixed-type LG-tub1

Group	Type of LG-tub1	Contained component	Abbreviations	Illustrations ^a^: Additional files: Figures S numbers	Colour	Mucin phenotype
Tub1	Pure	No other cancerous component.	LG-tub1		Whitish	Intestinal (I)
	Mixed-type	HG-tub1	LG-tub1 > HG-tub1		Isochromatic (but partly slightly reddish)	Gastrointestinal (GI)
LG-tub1 > tub2	Mixed-type	LG-tub2	LG-tub1 > LG-tub2		Whitish	Intestinal (I)
	Mixed-type	HG-tub2	LG-tub1 > HG-tub2		Reddish	Gastric (G)
	Mixed-type	HG-tub2 and por2.	LG-tub1 > HG-tub2-por2		Isochromatic	Gastric (G)

*LG-tub1* low-grade, well-differentiated tubular adenocarcinoma; *HG-tub1* high-grade, well-differentiated tubular adenocarcinoma; *LG-tub2* low-grade, moderately differentiated tubular adenocarcinoma; *HG-tub2* high-grade, moderately differentiated tubular adenocarcinoma; *LG-tub1 > HG-tub1* LG-tub1 containing HG-tub1; *LG-tub1 > LG-tub2* LG-tub1 containing LG-tub2; *LG-tub1 > HG-tub2* LG-tub1 containing HG-tub2; *Por2* non-solid type, poorly differentiated adenocarcinoma; *LG-tub1 > HG-tub2-por2* LG-tub1 containing HG-tub2 with por2. ^a^In the illustration of the LG-tub1 > HG-tub1, some parts were revised and transferred from the reference [8] by permission of the copyright holder (TS: the corresponding author of this article)

The endoscopic and clinicopathological characteristics of all 16 intramucosal histologically mixed-type LG-tub1 lesions are listed in Table 5. The largest lesion in the LG-tub1 > tub2 group exhibited metachronous lesions, a 0-IIb appearance, was isochromatic and had a G-phenotype. This lesion was the third largest of all 60 LG-tub1 lesions. Histopathological examination of the post-ESD specimen revealed an area in this lesion in which the HG-tub2 and a minor undifferentiated (non-solid, poorly differentiated adenocarcinoma [por2]) component present in a mixed fashion, which could not be measured separately [3]. However, this area was less than 20 mm (illustrations of LG-tub1 > HG-tub2 with por2 [LG-tub1 > HG-tub2-por2] are presented in Table 4). The patient with this lesion had undergone endoscopic resection of metachronous lesions > 1 year prior to the current study. Because this was the only lesion with a minor undifferentiated component, multivariate logistic regression analysis could not be performed.

**Table 5:** List of histologically mixed-type, LG-tub1 lesions

Case number	Atypia grade-histology contained in LG-tub1	Age	Sex (M/F)	Maximum diameter (mm)	Macroscopic type (0-IIa/0-IIb/0-IIc^a^)	Color on WLE (W/Iso/R)	DL on NBI-ME	Mucin phenotype (G/GI/I)	*H. pylori* status	Endoscopically resected metachronous EGC lesion > 1 year before this study
1	HG-tub2-por2	93	M	31	0-IIb	Iso	Clear	G	–	+
2	HG-tub2	80	M	15	0-IIa	R	Clear	G	+	+
3	HG-tub2	57	M	12	0-IIc	R	Clear	G	+	–
4	HG-tub2	62	M	10	0-IIc	W	Clear	G	+	–
5	HG-tub2	69	M	8	0-IIb	R	Clear	GI	+	–
6	HG-tub2	91	M	14	0-IIc	R	Clear	I	–	+
7	LG-tub2	80	F	17	0-IIc	W	Clear	I	+	–
8	HG-tub1	68	M	14	0-IIa	W	Clear	G	–	–
9	HG-tub1	73	M	11	0-IIa	W	Clear	G	+	–
10	HG-tub1	81	M	6	0-IIa	W	Clear	G	–	–
11	HG-tub1	77	M	70	0-IIb	Iso	Partly unclear	GI	–	+
12	HG-tub1	65	M	43	0-IIb	Iso	Partly unclear	GI	+	–
13	HG-tub1	77	M	14	0-IIc	R	Clear	GI	–	–
14	HG-tub1	67	F	10	0-IIa	W	Clear	I	+	–
15	HG-tub1	73	F	8	0-IIa	W	Clear	I	+	–
16	HG-tub1	70	M	2	0-IIb	Iso	Clear	I	–	–

*LG-tub1* low-grade, well-differentiated tubular adenocarcinoma; *M* male; *F* female; *U* upper-third; *M* middle-third; *L* lower-third portion of the stomach; *WLE* white light endoscopy; *W* whitish; *Iso* isochromatic; *R* reddish; *DL* demarcation line; *NBI-ME* narrow-band imaging with magnifying endoscopy; *G* gastric; *GI* gastro-intestinal; *I* intestinal mucin phenotype; *EGC* early gastric cancer; *HG* high-grade; *Tub2* moderately differentiated tubular adenocarcinoma; *Por2* non-solid type, poorly differentiated adenocarcinoma; ^a^*0-IIa* superficial elevated; *0-IIb* superficial flat; *0-IIc* superficial depressed type [3]

Six of the 60 LG-tub1 lesions met the expanded indications for ESD, and thus, the endoscopic and clinicopathological features of these six LG-tub1 lesions were compared (Table 6). All three histologically pure LG-tub1 lesions exhibited a 0-IIa appearances, a whitish colour, clear DLs on NBI-ME, and had I-phenotypes. Conversely, all three histologically mixed-type LG-tub1 lesions exhibited a 0-IIb appearance and an isochromatic colour. Two of these histologically mixed-type LG-tub1 lesions showed partly unclear DLs even on NBI-ME due to non-cancerous epithelia interjacent at tumour DLs, had GI-phenotypes, and did not have histologically mixed (a) minor undifferentiated component(s) despite their larger diameters.

**Table 6:** List of LG-tub1 lesions measuring more than 20 mm with indications for ESD

Case number (same as Table 5)	Pure/mixed component	Age	Sex (M/F)	Maximum diameter (mm)	Macroscopic type (0-IIa/0-IIb/0-IIc^a^)	Colour on WLE (W/Iso/R)	Background mucosal atrophy (C-I, II, III, O-I, II, III^b^)	DL on NBI-ME	*H. pylori* status	Mucin phenotype (G/GI/I)
1	Pure	60	M	27	0-IIa	W	O-I	Clear	+	I
2	Pure	79	F	26	0-IIa	W	O-III	Clear	–	I
3	Pure	68	M	24	0-IIa	W	O-III	Clear	+	I
4 (11)	HG-tub1	77	M	70	0-IIb	Iso	O-III	Partly unclear	–	GI
5 (12)	HG-tub1	65	M	43	0-IIb	Iso	O-II	Partly unclear	+	GI
6 (1)	HG-tub2-por2	93	M	31	0-IIb	Iso	O-III	Clear	–	G

*LG-tub1* low-grade, well-differentiated tubular adenocarcinoma; *ESD* endoscopic submucosal dissection; *M* male; *F* female; *U* upper-third; *M* middle-third; *L* lower-third portion of the stomach; *WLE* white light endoscopy; *W* whitish; *Iso* isochromatic; *R* reddish; *DL* demarcation line; *NBI-ME* narrow-band imaging with magnifying endoscopy; *G* gastric; *GI* gastrointestinal; *I* intestinal mucin phenotype; *HG* high-grade; *Tub2* moderately differentiated tubular adenocarcinoma; *Por2* non-solid type, poorly differentiated adenocarcinoma; ^a^*0-IIa* superficial elevated; *0-IIb* superficial flat; *0-IIc* superficial depressed type [3]; ^b^Kimura-Takemoto classification [24]

### Clinical characteristics

The clinical characteristics of the patients in the tub1 and LG-tub1 > tub2 groups were compared (Table 3), and no significant differences were found in the age, sex, or *H. pylori* status. Six of the 60 LG-tub1s were detected in the stomach of patients who had successfully eradicated an *H. pylori* infection. All of the six LG-tub1s belonged to the tub1 groups (4 pure LG-tub1 lesions and 2 LG-tub1 > HG-tub1 lesions). The frequency of the metachronous EGC lesions that were resected > 1 year prior to the study was significantly higher in the LG-tub1 > tub2 group than in the tub1 group (*P* = 0.009).

## Discussion

Generally, the higher the atypia grade and the lower the degree of differentiation that tumours exhibit, the greater their malignant characteristics. LG-tub1 lesions have the lowest atypia grade and differentiation degree among the differentiated-type EGCs. It is suggested that an intramucosal pure LG-tub1 lesion, in proportion to its diameters, may be able to transform into a tumour with a higher atypia grade and lower differentiation degree and grow to contain (a) minor undifferentiated component(s), ultimately acquiring more malignant characteristics. Accordingly, LG-tub1s may be considered an important type of EGCs given the growth of differentiated-type gastric cancers [7, 8]. In other words, these changes in LG-tub1 lesions may occur through the following processes.

First, the pure LG-tub1 lesions form at the beginning of differentiated-type gastric cancer development. Second, a minor portion of the LG-tub1 lesion may subsequently change into an HG-tub1 component and the LG-tub1 lesion may be diagnosed as an LG-tub1 > HG-tub1 lesion. Third, the HG-tub1 component of the LG-tub1 > HG-tub1 lesion may grow and become a lower differentiation degree component HG-tub2 and be diagnosed as LG-tub1 > HG-tub2. Fourth, the LG-tub1 > HG-tub2 lesion might grow up to contain (a) minor undifferentiated component(s). The fourth process in the changes in LG-tub1 lesions, which are included in differentiated-type EGCs, has been reported to occur more frequently in LG-tub1 lesions with G-phenotype than in those with other-mucin-phenotype [9–13]. Accordingly, it is also suggested that the frequency of the intramucosal G-phenotype LG-tub1 > HG-tub2 without (a) minor undifferentiated component(s) growing to contain (a) minor undifferentiated component(s) in proportion to their diameter is higher than that of other-mucin-phenotype LG-tub1 > HG-tub2 lesions without (a) minor undifferentiated component(s).

The largest lesion with por2 in the LG-tub1 > tub2 group might have grown to infiltrate the lymph vessels, eventually leading to lymph node metastasis, if it had been overlooked due to diagnostic difficulty resulting from its 0-IIb appearance and isochromatic colour. To confirm that the G-phenotype LG-tub1 > HG-tub2 lesions grow to contain (a) minor undifferentiated component(s), consequently infiltrating the lymph vessels and resulting in lymph node metastasis, comparisons between these G-phenotype LG-tub1 > HG-tub2 lesions without and with these three factors described above are needed. However, it is very difficult to conduct such comparisons in the clinical setting because the glandular architecture and cytological atypia grade change from LG to HG in proportion to diameter [4, 8], which suggests that a considerable number of LG-tub1 cells convert to HG-tub1 cells and that LG-tub1 > HG-tub2 lesions are diagnosed as HG-tub1 > HG-tub2 (synonymous with predominant HG-tub1 containing HG-tub2) lesions when the intramucosal G-phenotype LG-tub1 > HG-tub2 lesions grow to induce lymphatic permeation and lymph node metastasis [4, 8–13].

Reportedly, differentiated-type EGC lesions have more microvessels and are more reddish than undifferentiated-type EGC lesions [26–28]. Additionally, the endoscopic colour of 0-IIb-type EGC is significantly correlated with the degree of tumour differentiation, the tumour size, the extent of wall infiltration, and number of microvessels [27]. Because the intramucosal G-phenotype LG-tub1 > HG-tub2 lesions presented with higher frequencies of the 0-IIc type and a reddish colour in this study, the diagnoses of the tumours using WLE may not be overly difficult. In contrast, the G-phenotype LG-tub1 > HG-tub2 lesions containing (a) minor undifferentiated component(s) and having larger diameters than the other LG-tub1 > HG-tub2 lesions without (a) minor undifferentiated component(s) (e.g., the largest lesion of the LG-tub1 > HG-tub2 group in this study) might be isochromatic and have a higher frequency of the 0-IIb type [27]. Therefore, if these lesions have a 0-IIb type appearance, they may be difficult to diagnose using only WLE despite their larger diameters compared with the LG-tub1 > HG-tub2 tumours without (a) minor undifferentiated component(s).

NBI-ME has been reported to be superior to WLE for assessing DLs and diagnosing possible histological subtypes of differentiated-type EGC lesions [8, 22, 29–32]. We have reported that the limited-to-four-pattern sign on NBI-ME can be used to distinguish intramucosal, histologically mixed-type LG-tub1 lesions from pure LG-tub1 lesions with high diagnostic accuracy (88.9%) and that some of these tumours have partly unclear DLs even on NBI-ME observation [8]. However, the mucin phenotypes, atypia grades, and histological subtypes of these tumours showing partly unclear DLs have not yet been reported. In this study, the two GI-phenotype LG-tub1 > HG-tub1 lesions showing partly unclear DLs even on NBI-ME, were larger than 30 mm and met the expanded indication for ESD (Table 4, Table 6, and Additional file 2: Figure S2). It was necessary to obtain negative biopsy specimens around these lesions to determine the area that required resection by ESD and avoid residual cancer due to partly unclear DLs. However, these LG-tub1 > HG-tub1 lesions did not contain (a) minor undifferentiated component(s) despite their larger diameters. These GI-phenotype LG-tub1 > HG-tub1 lesions might have difficulty growing to contain (a) minor undifferentiated component(s). Additionally, NBI-ME may be less accurate in determining combinations of histological atypia grades and subtypes (e.g., HG-tub2) that are present in the histologically mixed-type LG-tub1 tumours, except for areas exhibiting typical NBI-ME patterns (e.g., cork-screw pattern [30] and the four patterns of LG-tub1 tumours [8]). Therefore, biopsy is required for precise determination of whether these mixed-type LG-tub1 lesions contain HG-tub2 and/or (a) minor undifferentiated component(s).

Regardless of the maximum diameter, intramucosal, differentiated-type predominant, histologically mixed-type EGCs which include G-phenotype LG-tub1 > HG-tub2 lesions, containing (a) minor undifferentiated component(s) whose diameters are 20 mm or less, meet the expanded indication for ESD [8, 14]. Reportedly, intramucosal, histologically mixed-type EGC lesions that measure ≤30 mm and have compositions of 67% tub2 and por2 components may not require further treatment after curative ESD because their risk of lymph node metastasis is found to be < 1% regardless of the presence of ulcerations [18, 19]. Intramucosal, differentiated-type predominant, histologically mixed-type EGCs that measure more than 20 mm, exhibit no ulceration and meet the expanded indications also reportedly have a good prognosis after ESD. However, it was reported that patients with intramucosal, differentiated-type predominant, histologically mixed-type EGCs of expanded indication, including G-phenotype LG-tub1 > HG-tub2 lesions, occasionally include patients who experience late-onset metastasis to lymph node and death from primary gastric cancer after ESD [17, 20]. The evidence is insufficient regarding whether intramucosal, G-phenotype LG-tub1 > HG-tub2 lesions that have diameters of more than 30 mm or diameters of more than 40 mm containing (a) minor undifferentiated component(s) whose diameters are more than 20 mm can be cured by ESD [14, 18–21]. Additionally, in some G-phenotype LG-tub1 > HG-tub2 lesions containing (a) minor undifferentiated component(s), it is occasionally difficult to diagnose the maximum diameter of the undifferentiated component(s). Nearly all the patients who undergo curative resection for intramucosal EGC lesions have a good prognosis [15–17]. However, clarifying the clinicopathological features of the G-phenotype LG-tub1 > HG-tub2 lesions to exactly diagnose and treat these patients is potentially important for gastric cancer screening, may help clinician carefully follow up with patients after ESD of these tumours, and might decrease the number of patients who experience late-onset lymph node metastasis [17, 20]. Moreover, enhancing the attention given to these tumours by not only endoscopists but also gastroenterologists, surgeons who perform ESD, and clinicians in other medical fields is necessary. In addition, some of the topics of this study may remind clinicians of the importance of stomach cancer screening.

Eradication of *H. pylori* has been reported to reduce the incidence of gastric cancer [33, 34]. However, unfortunately, it has recently been reported that gastric cancer can occur in the patients from whom *H. pylori* was successfully eradicated [34, 35]. Therefore, careful attention to EGC (including LG-tub1) that may occur in patients after successful eradication of *H. pylori* is necessary. The objects of this study were LG-tub1 tumours. There was not a significant difference in the frequency of active *H. pylori* infection between the tub1 and LG-tub1 > tub2 groups. However, six of the 60 LG-tub1 lesions examined consisted of the 4 pure LG-tub1s and the 2 LG-tub1 > HG-tub1 tumours, belonged to the tub1 group, and were detected in patients with successful *H. pylori* eradication. Accordingly, these results might support our supposition about the third process of the changes in LG-tub1 lesions as described above. In other words, persistent infection of *H. pylori* might be necessary to the third process. Moreover, these processes of changes in LG-tub1 lesions may occur even after successful *H. pylori* eradication in the stomachs. However, why these processes occur after successful *H. pylori* eradication is not yet clear. To confirm these processes clinically, it is necessary for us to examine a larger number of LG-tub1 tumours that are divided into an active *H. pylori* infected group and successfully *H. pylori*-eradicated group. However, it is also very difficult to conduct such comparisons in the clinical setting. The reason for the difficulty was described above.

## Conclusions

Among the intramucosal, histologically mixed-type LG-tub1 lesions, G-phenotype LG-tub1 > HG-tub2 lesions might grow to contain (a) minor undifferentiated component(s) in proportion to their diameters and have a higher risk of causing late-onset lymph node metastasis than other-mucin-phenotype LG-tub1 > HG-tub2, LG-tub1 > HG-tub1, and LG-tub1 > LG-tub2 lesions. The clinicopathological features of intramucosal G-phenotype LG-tub1 > HG-tub2 lesions may be specified as superficial depressed in shape and reddish colour. However, these tumours with (a) minor undifferentiated component(s) might change form reddish to isochromatic. Careful attention should be paid to LG-tub1 lesions with the potential to change into risky G-phenotype LG-tub1 > HG-tub2 lesions because the lesions might grow to contain (a) minor undifferentiated component(s) and cause late-onset lymph node metastasis after ESD. Exactly diagnosing and treating risky G-phenotype LG-tub1 > HG-tub2 lesions may help clinicians carefully follow up patients with these tumours after ESD and might decrease the number of patients who experience late-onset lymph node metastasis.

## Additional files

Additional file 1: **Figure S1.** Endoscopic and histopathological findings of a superficial elevated type (0-IIa), I-phenotype pure LG-tub1 lesion. The white arrows and cautery markings surround the lesions. The yellow dotted lines indicate the demarcation lines (DLs). The microphotographs of the resected specimens were stained with haematoxylin-eosin (HE). (JPG 1063 kb)

Additional file 2: **Figure S2.** Endoscopic and histopathological findings of a superficial flat type (0-IIb), GI-phenotype, LG-tub1 > HG-tub1 lesion. The white arrows and cautery markings surround the lesions. The yellow dotted lines indicate the demarcation lines (DLs). The green and red solid gentle curves indicate a LG-tub1 > HG-tub1 tumour. The microphotographs of the resected specimens were stained with haematoxylin-eosin (HE). Some parts were revised and transferred from the reference [8] by permission of the copyright holder (TS: the corresponding author of this article). (JPG 1670 kb)

Additional file 3: **Figure S3.** Endoscopic and histopathological findings of a superficial shallow depressed type (0-IIc), I-phenotype, LG-tub1 > LG-tub2 lesion. The white arrows surround the lesions. The yellow dotted lines indicate the demarcation lines (DLs). The microphotographs of the resected specimens were stained with haematoxylin-eosin (HE). (JPG 1563 kb)

Additional file 4: **Figure S4.** Endoscopic and histopathological findings of a superficial depressed type (0-IIc), G-phenotype LG-tub1 > HG-tub2 lesion. The white arrows surround the lesions. The yellow dotted line indicates the demarcation lines (DLs). The microphotographs of the resected specimens were stained with haematoxylin-eosin (HE). The black arrows indicate the carcinomatous part. (JPG 1814 kb)

Additional file 5: **Figure S5.** Endoscopic and histopathological findings of a superficial flat type (0-IIb), G-phenotype LG-tub1 > HG-tub2 lesion. The white arrows and cautery markings surround the lesions. The yellow dotted lines indicate the demarcation lines (DLs). The microphotographs of the resected specimens were stained with haematoxylin-eosin (HE). The solid red lines show G-phenotype LG-tub1s and the broken red lines show G-phenotype HG-tub2s mixed with por2s (LG-tub1 > tub2-por2s) in a post-endoscopic submucosal dissection (ESD) specimen. Moderately magnified microphotograph of a haematoxylin-eosin (HE)-stained section of a deeper cut of the number 9b slice in the post-ESD specimen revealed that the LG-tub1 tumour was adjacent to a HG-tub2-por2 tumour in this single LG-tub1 > HG-tub2-por2 lesion. (JPG 1791 kb)

## Abbreviations

DL: Demarcation line
EGC: Early gastric cancer
ESD: Endoscopic submucosal dissection
G: Gastric
GI: Gastrointestinal
HE: Haematoxylin-eosin
HG: High-grade
HG-tub1: HG, well-differentiated tubular adenocarcinoma
HG-tub2: HG, moderately differentiated tubular adenocarcinoma
I: Intestinal
LG: Low-grade
LG-tub1 > HG-tub2: LG-tub1 containing HG-tub2
LG-tub1 > HG-tub2-por2: LG-tub1 containing HG-tub2 with non-solid type, poorly differentiated adenocarcinoma
LG-tub1 > tub2: LG-tub1 containing LG-tub2 or HG-tub2
LG-tub1: LG, well-differentiated tubular adenocarcinoma
LG-tub2: LG, moderately differentiated tubular adenocarcinoma
N: Null mucin phenotype
NBI-ME: Narrow band imaging with magnifying endoscopy
Por2: Non-solid type, poorly differentiated adenocarcinoma
SM: Submucosa WLE: White light endoscopy
