# Supplementary figures and images for: Endoscopic and clinicopathological features of intramucosal, histologically mixed-type, low-grade, well-differentiated gastric tubular adenocarcinoma with the potential for late-onset lymph node metastasis
Source: BMC Gastroenterol. 2018 Dec 27;18:189. doi: 10.1186/s12876-018-0919-3 (PMC6307236; doi:10.1186/s12876-018-0919-3)

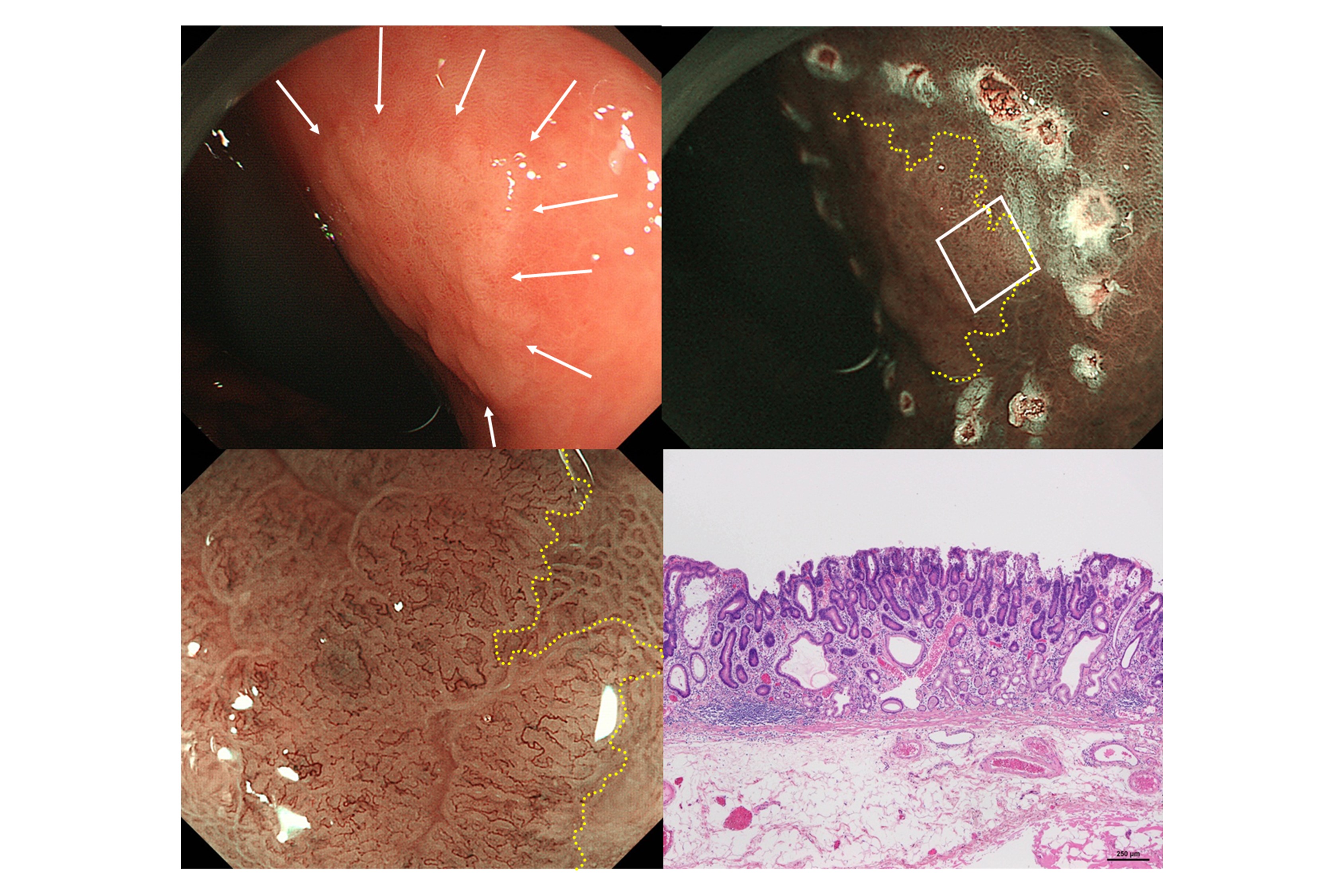

Supplement: Supplementary file 1 — Figure S1. Endoscopic and histopathological findings of a superficial elevated type (0-IIa), I-phenotype pure LG-tub1 lesion. The white arrows and cautery markings surround the lesions. The yellow dotted lines indicate the demarcation lines (DLs). The microphotographs of the resected specimens were stained with haematoxylin-eosin (HE). (JPG 1063 kb) [file 12876_2018_919_MOESM1_ESM.jpg]

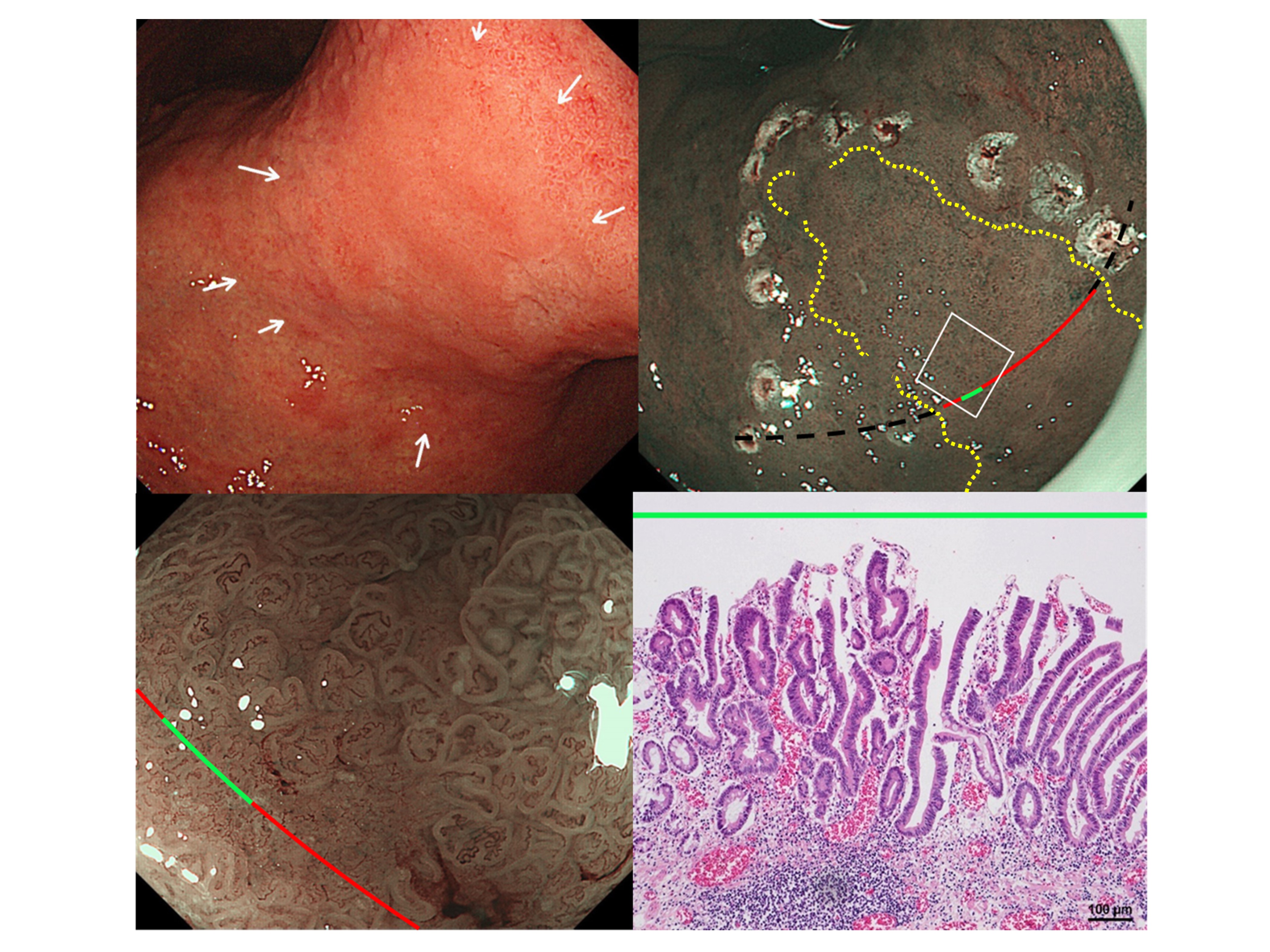

Supplement: Supplementary file 2 — Figure S2. Endoscopic and histopathological findings of a superficial flat type (0-IIb), GI-phenotype, LG-tub1 > HG-tub1 lesion. The white arrows and cautery markings surround the lesions. The yellow dotted lines indicate the demarcation lines (DLs). The green and red solid gentle curves indicate a LG-tub1 > HG-tub1 tumour. The microphotographs of the resected specimens were stained with haematoxylin-eosin (HE). Some parts were revised and transferred from the reference [8] by permission of the copyright holder (TS: the corresponding author of this article). (JPG 1670 kb) [file 12876_2018_919_MOESM2_ESM.jpg]

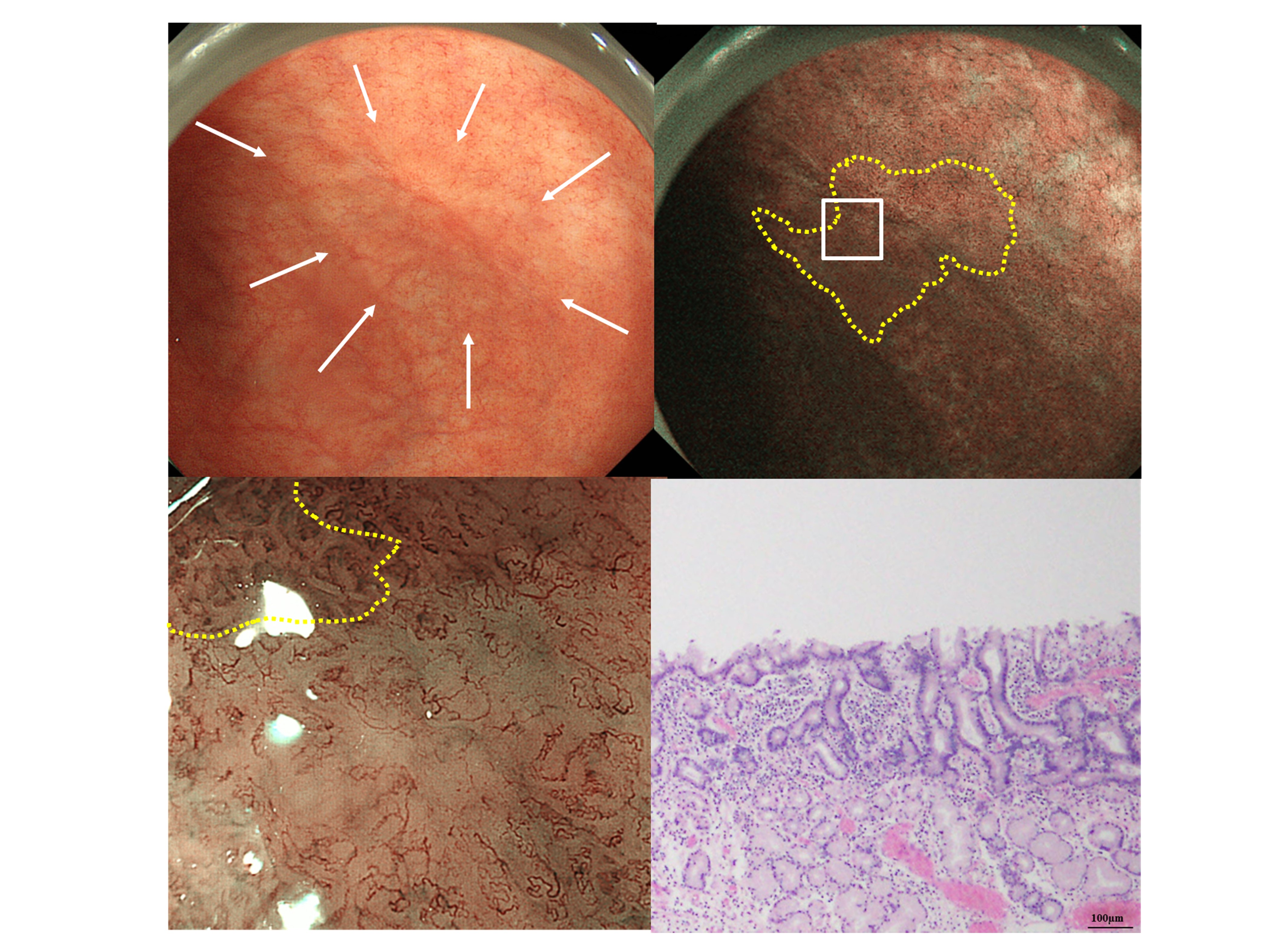

Supplement: Supplementary file 3 — Figure S3. Endoscopic and histopathological findings of a superficial shallow depressed type (0-IIc), I-phenotype, LG-tub1 > LG-tub2 lesion. The white arrows surround the lesions. The yellow dotted lines indicate the demarcation lines (DLs). The microphotographs of the resected specimens were stained with haematoxylin-eosin (HE). (JPG 1563 kb) [file 12876_2018_919_MOESM3_ESM.jpg]

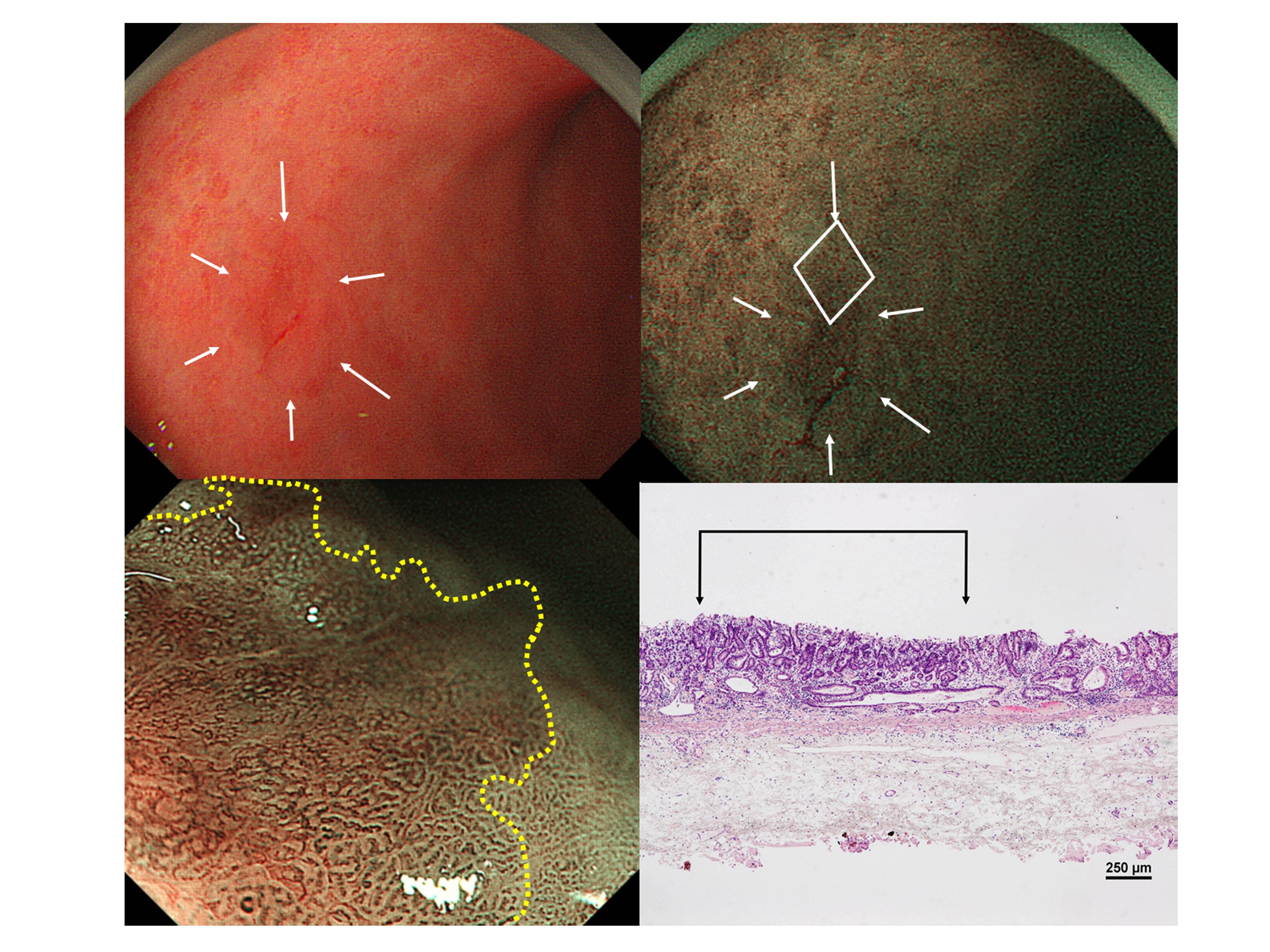

Supplement: Supplementary file 4 — Figure S4. Endoscopic and histopathological findings of a superficial depressed type (0-IIc), G-phenotype LG-tub1 > HG-tub2 lesion. The white arrows surround the lesions. The yellow dotted line indicates the demarcation lines (DLs). The microphotographs of the resected specimens were stained with haematoxylin-eosin (HE). The black arrows indicate the carcinomatous part. (JPG 1814 kb) [file 12876_2018_919_MOESM4_ESM.jpg]

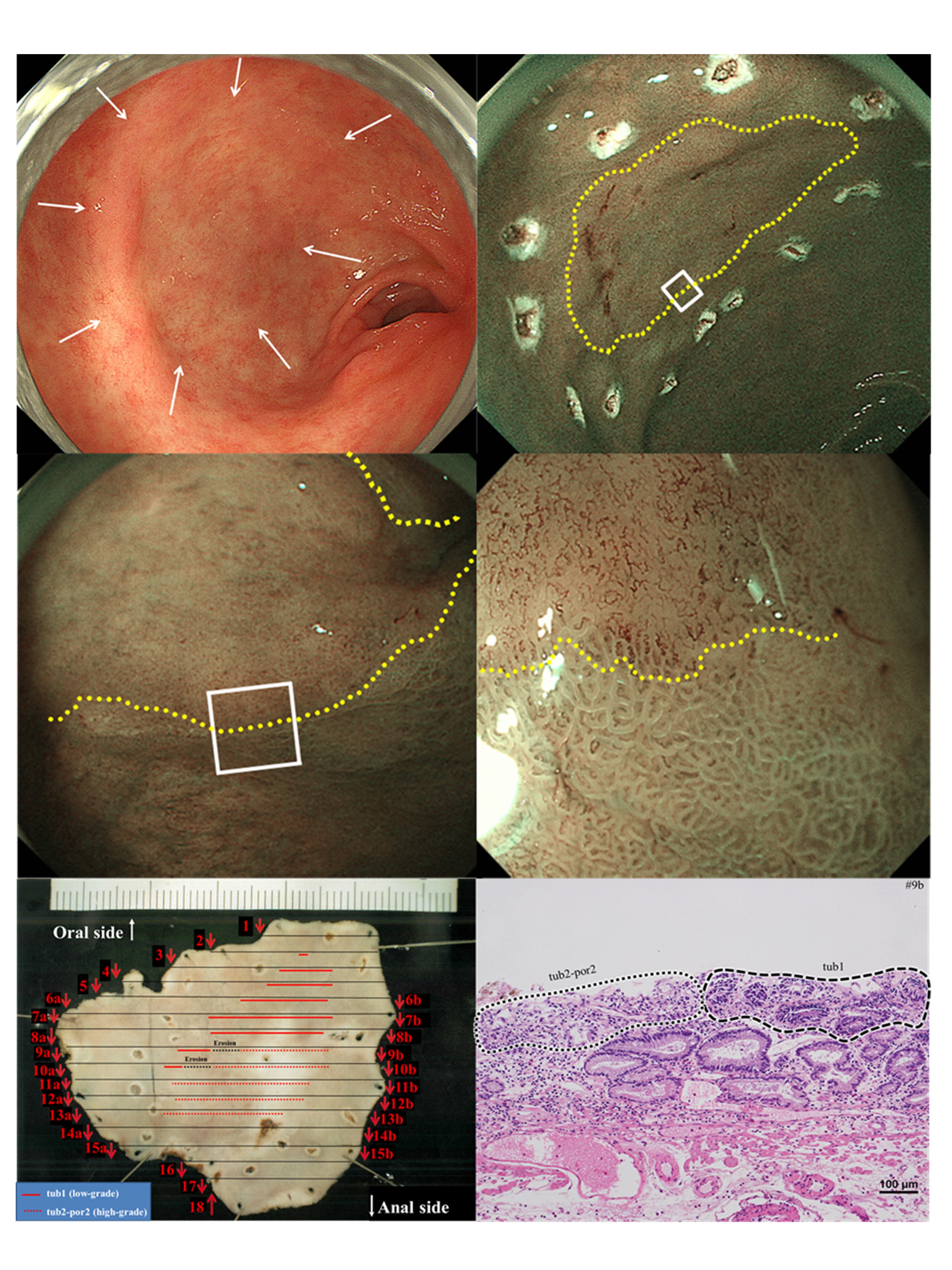

Supplement: Supplementary file 5 — Figure S5. Endoscopic and histopathological findings of a superficial flat type (0-IIb), G-phenotype LG-tub1 > HG-tub2 lesion. The white arrows and cautery markings surround the lesions. The yellow dotted lines indicate the demarcation lines (DLs). The microphotographs of the resected specimens were stained with haematoxylin-eosin (HE). The solid red lines show G-phenotype LG-tub1s and the broken red lines show G-phenotype HG-tub2s mixed with por2s (LG-tub1 > tub2-por2s) in a post-endoscopic submucosal dissection (ESD) specimen. Moderately magnified microphotograph of a haematoxylin-eosin (HE)-stained section of a deeper cut of the number 9b slice in the post-ESD specimen revealed that the LG-tub1 tumour was adjacent to a HG-tub2-por2 tumour in this single LG-tub1 > HG-tub2-por2 lesion. (JPG 1791 kb) [file 12876_2018_919_MOESM5_ESM.jpg]
